# Trends in accident-related traumatic dental injuries among children: a 10-year retrospective study of patients attending a university clinic

**DOI:** 10.1007/s00784-025-06546-4

**Published:** 2025-09-15

**Authors:** Anika Islam, Spyridon N. Papageorgiou, Blend Hamza

**Affiliations:** Clinic of Orthodontics and Pediatric Dentistry, Center for Dental Medicine, University of Zurich, Zurich, Switzerland

**Keywords:** Traumatic dental injury (TDI), Pediatric dentistry, Tooth fracture, Dentition stage

## Abstract

**Objective:**

This study aimed to analyze accident-related traumatic dental injury (TDI) data to determine the occurrence, characteristics, and types of dental injuries in primary and permanent dentitions of underage patients attending a university clinic.

**Materials and methods:**

A retrospective observational study was conducted at the Clinic of Orthodontics and Pediatric Dentistry of Zurich from 2010 to 2019 and involved children with accident-related TDIs from the ages of 0 to 18 years old. Data regarding patient- and trauma-related characteristics were extracted and analyzed statistically according to dentition, tooth, age, sex, and time elapsed before visiting the dentist at 5%.

**Results:**

The sample included 1,291 TDIs seen in a sample predominantly comprised of boys (59.8%), with a median age of 3.0 years (interquartile range 1.8–7.1 years), mostly in the deciduous dentition (71.6%) with ≥ 1 TDIs between 2010 and 2019. Accidents mainly occurred at home (45.6%) or outdoors (30.3%) and were primarily the result of falls (37.2%) or playtime (29.5%). Affected children often visited the dentist within the first 24 h (77.1%). Deciduous teeth (51.9%) and permanent teeth (37.2%; *p* = 0.001) primarily exhibited injuries to the periodontal tissue.

**Conclusion:**

This study found boys were more prone to TDI than girls. Significant differences were seen in the injury mechanisms between deciduous and permanent teeth. Moreover, injuries to the periodontal tissue, with or without tooth fractures, were the most common trauma found.

**Clinical relevance:**

Gaining insights into TDI patterns over time can help clinical practitioners develop more effective prevention measures.

**Supplementary Information:**

The online version contains supplementary material available at 10.1007/s00784-025-06546-4.

## Introduction

Traumatic dental injuries (TDIs) involve impact injury to the teeth or the adjacent hard/soft tissues within the oral cavity or around the vicinity of the mouth that often happen suddenly, unexpectedly, and accidentally. TDI prevalence varies considerably, depending on its definition and patient demographics, with an average prevalence between 18.1 and 24.2% [1, 2]. TDI incidence seems to increase during childhood up to approximately 12 years of age and then declines, with boys being more affected than girls [2–6]. As far as habits and everyday activities are concerned, sports activities have been identified as one of the major sources of TDI, especially contact sports such as boxing, karate, American football, rugby, basketball, hockey, soccer, cricket, and baseball [5, 7–9]. Morphologically, children with increased overjet, inadequate lip coverage, and open bite are more prone to TDIs, while a history of previous dental injuries is associated with recurrent traumatic episodes [2, 6, 10–12] .

The severity level of TDIs can range from minimal enamel cracks to a complex fracture that exposes the dental pulp or from subluxation to complete avulsions [2, 13]. The most, , commonly injured teeth are the upper central incisors in the primary and permanent dentitions [2, 14–17]. TDIs can have several consequences for patients and their families in the short, intermediate, and long terms, including pain, compromised esthetics, loss of or damage to hard/soft tissues, functional impairment, a need for multiple treatment procedures, and a reduced quality of life [5, 8, 18, 19]. Moreover, dental trauma has been shown to have significant financial and social burdens that are greater for more complex injuries and can span for decades for children, their legal guardians, and health service funders [20, 21]. Given that over 1 billion people have had TDIs, now the fifth most prevalent disease or injury worldwide [1, 22], efforts should be made to reduce this burden for patients, their families, and health services.

Even though several protocols for TDI management have been reported in recent Cochrane reviews [23] and clinical guidelines [24–27], efforts to prevent TDIs or reduce their impact may be more efficient. Therefore, it is important to raise awareness about the risk and impact of TDIs [9], the use of mouthguards to reduce their impact in high-intensity contact sports [28, 29], and orthodontic treatment to retract protruded front teeth and increase their coverage by the lips [6].

In conclusion, TDIs pose a challenge to the dental and overall health of children and adolescents. Awareness among parents, children, schoolteachers, and dental practitioners should be increased [24–27]. Such efforts can support the development or refinement of protocols for the effective prevention and management of TDIs, potentially reducing the associated socioeconomic burden. Therefore, this retrospective study aimed to assess the prevalence of TDIs and analyze differences according to patient demographics, injury type, number of affected teeth, location, and causes in Zurich, Switzerland, between 2010 and 2019.

## Materials and methods

This retrospective cross-sectional study was based on the archival data of patients treated at the Clinic of Orthodontics and Pediatric Dentistry of Zurich, Switzerland, from January 2010 to December 2019. After ethical approval (BASEC-Nr: 2025 − 00682), the files of all patients younger than 18 years attendng the university’s pediatric dentistry clinic for TDIs were identified and included. In an effort to increase generalizability and give a broad overview of TDIs, the inclusion criteria were (i) the presence of a TDI on any tooth, in either primary or permanent dentition as a consequence of an accident, as reported by the patient and the parent or legal guardian, and (ii) the patient being under 18 years old at the time of the TDI. The exclusion criterion was the absence of sufficient or complete records. No additional exclusions were made based on systemic health conditions, in order to reflect a real-world clinical population. As standard practice, the parents or legal guardians of all patients under 18 years old treated in the clinic sign an appropriate informed consent form before commencing diagnosis and treatment. These 10 years of data on dental injuries were registered by 16 postgraduates during their training in pediatric dentistry. To guarantee uniformity in diagnostic criteria and data recording methods, all 16 postgraduate examiners underwent standardized training facilitated by one highly skilled supervisor. The supervisor offered detailed, practical instruction, and all examiners were calibrated under his direct supervision to ensure consistency in data collection. Based on the total number of patients (*N* = 1,324), 1,237 patients were included in the study. TDI diagnosis was based on patient history and clinical examination, while intraoral/extraoral photographs and radiographs (periapical, panoramic, or cone-beam computerized tomography) were employed on occasion, according to the clinician’s judgement but not on a standard basis. All data was extracted manually by one investigator (AI) as part of her doctoral thesis, while another investigator (SNP) independently checked a randomly selected sample of 200 patient files for accuracy.

In accordance with the department standards, this study employed the classification system of TDI as proposed by the World Health Organization, with modification based on the recommendations of Andreasen et al. [30]. TDIs were categorized into three main groups: (1) injuries to the dental hard tissues and the pulp-including enamel infraction, enamel fracture, enamel-dentin fracture (uncomplicated crown fracture), enamel-dentin fracture with pulp exposure (complicated crown fracture), crown-root fracture (uncomplicated + complicated crown-root fracture), and root fractur; (2) injuries to the periodontal tissues-including concussion, subluxation, lateral luxation, intrusive luxation, extrusive luxation and avulsion; and (3) combined injuries, defined as the simultaneous occurrence of trauma to both dental hard tissue and the periodontal tissue in the same tooth, resulting from a single traumatic incident.

The following information was collected: patient characteristics (i.e., age and sex) and accident characteristics (i.e., date, accident location and cause, the time elapsed before the dental appointment, injury type, and affected teeth).

No sample size calculation was performed since all available patients with complete documentation who attended the university clinic from 2010 to 2019 were included (i.e., convenience sampling). Since over 1,000 patients were expected to be included, the study was deemed to have adequate statistical power.

The data were analyzed in an explorative fashion to identify trends and factors associated with TDIs. Data normality was checked through a visual inspection of distribution plots and a formal Shapiro–Wilk test. Descriptive statistics for non-normally distributed continuous variables included medians and interquartile ranges (IQRs). Differences between patient subgroups were assessed with the Kruskal–Wallis test for continuous variables and the chi-squared test (or Fisher’s exact test, if needed) for categorical variables. Furthermore, logistic regression was used to report odds ratios (ORs) with 95% confidence intervals (CIs) and appropriate interaction terms. Agreement between the two investigators performing data extraction was checked with the concordance correlation coefficient. All analyses were run in Stata (version 18.5; Stata Corp, College Station, TX) with double-sided α = 5% and an openly provided dataset (DOI: 10.5281/zenodo.14047897).

## Results

### Demographics overview

The sample included 1,291 injury cases of patients who had at least one reported TDI and attended the university pediatric dentistry clinic in Zurich between 2010 and 2019 (Tables 1, 2, 3, 4, 5, 6, 7, 8, 9 and 10). A total of 6,011 (4,112 deciduous teeth and 1,899 permanent) teeth were affected by traumatic injuries (Appendix 1). Observed injuries were predominantly seen in male patients (59.8%) deciduous dentition (71.60%). These patients had a median age of 3.0 years (IQR 1.8–7.1 years; range 1.7 months–18.0 years). Most were 0–6.0 years old (*n* = 920; 71.2%), followed by those who were 6.1–12.0 years old (*n* = 306; 23.7%), and those 12.1–18.0 years old (*n* = 65; 5.0%; Table 1). The majority of injuries were observed in patients in deciduous dentition (*n* = 924; 71.6%), followed by those with permanent dentition (*n* = 312; 24.2%), and those with mixed dentition (*n* = 55; 4.3%; Table 1). Patients who were between 0 and 6.0 years old predominantly had injuries to the periodontal tissue (47.0%), while those 6.1–12.0 and 12.1–18.0 years old mostly experienced injuries to the hard dental tissues and the pulp, alone or together with injuries to the periodontal tissue (63.3% and 79.4%, respectively; Table 9; *p* < 0.001). TDIs were relatively well distributed by month, with the highest prevalence in October (10.3%) and the lowest in February and September (7.1%; Table 1). Accidents were reported most often on Thursdays (16.5%) and least often on Saturdays (11.3%; Table 1; Appendix 2; the department is closed during weekends). Among the 1,237 affected patients, 1,187 (96.0%) had one injury, 44 (3.6%) had two injuries, 4 (0.3%) had three injuries, and one (0.1%) had four injuries (Table 10). Patients who attended the university clinic within 24 h of an accident accounted for 59.2% of the cases (Table 1). Among these, patients presented most frequently after injuries to the hard dental tissues and the pulp (44.7%), followed by injuries to the periodontal tissue (31.1%; *p* < 0.001; Table 9). Accidents were reported to have occurred mostly at home (primary place of residence and indoor and outdoor areas of the home: living room, kitchen, bedroom, bathroom etc.) (45.6%), followed by outdoors (30.3%), at school (school grounds: classroom, playgrounds, gymnasium, hallways) (7.9%), at preschool (7.5%), on a playground (5.0%), and in a swimming pool (3.7%; Appendix 3). Patients reported that falls mostly comprised their accidents (37.2%), followed by playtime (29.5%), sports (8.0%), bicycle riding (7.9%), traffic accidents (6.5%), falling downstairs (5.8%), and scooter use (5.2%; Appendix 4).

**Table 1:** Characteristics of the analyzed sample

Variable	Category	Metric	Frequency
Gender (*N* = 1291)	Female	n (%)	519 (40.2%)
	Male	n (%)	772 (59.8%)
Dentition stage (*N* = 1291)	Deciduous	n (%)	924 (71.6%)
	Mixed	n (%)	55 (4.3%)
	Permanent	n (%)	312 (24.2%)
Age (years) (*N* = 1291)		Median (IQR) [range]	3.0 (1.8, 7.1) [0.1, 18.0]
Age-group (*N* = 1291)	0–6.0 years	n (%)	920 (71.2%)
	6.1–12.0 years	n (%)	306 (23.7%)
	12.1–18.0 years	n (%)	65 (5.0%)
Accident year (*N* = 1291)	2010	n (%)	161 (12.5%)
	2011	n (%)	183 (14.2%)
	2012	n (%)	123 (9.5%)
	2013	n (%)	119 (9.2%)
	2014	n (%)	110 (8.5%)
	2015	n (%)	165 (12.8%)
	2016	n (%)	131 (10.2%)
	2017	n (%)	117 (9.1%)
	2018	n (%)	110 (8.5%)
	2019	n (%)	72 (5.6%)
Accident month (*N* = 1291)	January	n (%)	94 (7.3%)
	February	n (%)	92 (7.1%)
	March	n (%)	107 (8.3%)
	April	n (%)	128 (9.9%)
	May	n (%)	119 (9.2%)
	June	n (%)	108 (8.4%)
	July	n (%)	107 (8.3%)
	August	n (%)	96 (7.4%)
	September	n (%)	91 (7.1%)
	October	n (%)	133 (10.3%)
	November	n (%)	110 (8.5%)
	December	n (%)	106 (8.2%)
Accident day (*N* = 1291)	Monday	n (%)	195 (15.1%)
	Tuesday	n (%)	198 (15.3%)
	Wednesday	n (%)	197 (15.3%)
	Thursday	n (%)	213 (16.5%)
	Friday	n (%)	186 (14.4%)
	Saturday	n (%)	146 (11.3%)
	Sunday	n (%)	156 (12.1%)
Number of accidents (*N* = 1237)	1	n (%)	1188 (96.0%)
	2	n (%)	44 (3.6%)
	3	n (%)	4 (0.3%)
	4	n (%)	1 (0.1%)
Time < 24 h (*N* = 1258)	No	n (%)	513 (40.8%)
	Yes	n (%)	745 (59.2%)
Accident place (*N* = 1127)	At home	n (%)	514 (45.6%)
	Outdoor	n (%)	341 (30.3%)
	Playground	n (%)	56 (5.0%)
	Swimming pool	n (%)	42 (3.7%)
	Preschool	n (%)	85 (7.5%)
	School	n (%)	89 (7.9%)
Accident cause (*N* = 1280)	Accident	n (%)	83 (6.5%)
	Bicycle	n (%)	101 (7.9%)
	Fall	n (%)	476 (37.2%)
	Fall of stairs	n (%)	74 (5.8%)
	Playtime	n (%)	377 (29.5%)
	Scooter	n (%)	67 (5.2%)
	Sport	n (%)	102 (8.0%)
Soft tissue trauma extraoral (*N* = 1291)	No	n (%)	796 (61.7%)
	Yes	n (%)	495 (38.3%)
Soft tissue trauma intraoral (*N* = 1291)	No	n (%)	715 (55.4%)
	Yes	n (%)	576 (44.6%)
Injury type (*N* = 1291)	Injuries to the periodontal tissues	n (%)	651 (50.4%)
	Injuries to the hard dental tissues and the pulp	n (%)	350 (27.1%)
	Combined injury (injuries to the periodontal tissues & injuries to the hard dental tissues and the pulp)	n (%)	283 (21.9%)
	Soft-tissue injury	n (%)	7 (0.5%)
Avulsion (*N* = 1291)	No	n (%)	1159 (89.8%)
	Yes	n (%)	132 (10.2%)
No of avulsed teeth (*N* = 132)	1	n (%)	102 (77.3%)
	2	n (%)	20 (15.2%)
	3	n (%)	6 (4.6%)
	4	n (%)	3 (2.3%)
	6	n (%)	1 (0.8%)
	Total	N	178
Lateral luxation (*N* = 1291)	No	n (%)	977 (75.7%)
	Yes	n (%)	314 (24.3%)
No of laterally luxated teeth (*N* = 314)	1	n (%)	164 (52.2%)
	2	n (%)	111 (35.4%)
	3	n (%)	19 (6.1%)
	4	n (%)	17 (5.4%)
	5	n (%)	1 (0.3%)
	6	n (%)	2 (0.6%)
	Total	N	528
Subluxation (*N* = 1291)	No	n (%)	1008 (78.1%)
	Yes	n (%)	283 (21.9%)
Number of subluxated teeth (*N* = 283)	1	n (%)	110 (38.9%)
	2	n (%)	121 (42.8%)
	3	n (%)	20 (7.1%)
	4	n (%)	24 (8.5%)
	5	n (%)	1 (0.4%)
	6	n (%)	1 (0.4%)
	7	n (%)	3 (1.1%)
	8	n (%)	3 (1.1%)
	Total	N	564
Concussion (*N* = 1291)	No	n (%)	342 (26.5%)
	Yes	n (%)	949 (73.5%)
No of concussed teeth - exact (*N* = 949)	1	n (%)	213 (22.4%)
	2	n (%)	269 (28.4%)
	3	n (%)	86 (9.1%)
	4	n (%)	126 (13.3%)
	5	n (%)	28 (3.0%)
	6	n (%)	86 (9.1%)
	7	n (%)	53 (5.6%)
	8	n (%)	68 (7.2%)
	≥ 9	n (%)	20 (2.1%)
No of concussed teeth (*N* = 949)	One	n (%)	213 (22.4%)
	>one	n (%)	736 (77.6%)
	Total	N	3465
Intrusive luxation (*N* = 1291)	No	n (%)	1120 (86.8%)
	Yes	n (%)	171 (13.3%)
No of intrusive luxated teeth (*N* = 171)	1	n (%)	97 (56.7%)
	2	n (%)	62 (36.3%)
	3	n (%)	3 (1.8%)
	4	n (%)	7 (4.1%)
	6	n (%)	1 (0.6%)
	7	n (%)	1 (0.6%)
	Total	N	271
Extrusive luxation (*N* = 1291)	No	n (%)	1249 (96.8%)
	Yes	n (%)	42 (3.3%)
No of extrusively luxated teeth (*N* = 42)	1	n (%)	31 (73.8%)
	2	n (%)	9 (21.4%)
	3	n (%)	1 (2.4%)
	4	n (%)	1 (2.4%)
	Total	N	56
Enamel fracture (*N* = 1291)	No	n (%)	1028 (79.6%)
	Yes	n (%)	263 (20.4%)
No of enamel fractured teeth (*N* = 263)	1	n (%)	169 (64.3%)
	2	n (%)	69 (26.2%)
	3	n (%)	15 (5.7%)
	4	n (%)	7 (2.7%)
	5	n (%)	1 (0.4%)
	6	n (%)	1 (0.4%)
	7	n (%)	1 (0.4%)
	Total	N	398
Uncomplicated crown fracture (*N* = 1291)	No	n (%)	1051 (81.4%)
	Yes	n (%)	240 (18.6%)
No of uncomplicated crown fractured teeth (*N* = 240)	1	n (%)	176 (73.4%)
	2	n (%)	48 (19.9%)
	3	n (%)	9 (3.7%)
	4	n (%)	6 (2.5%)
	5	n (%)	1 (0.4%)
	Total	N	328
Complicated crown fracture (*N* = 1291)	No	n (%)	1101 (85.3%)
	Yes	n (%)	190 (14.7%)
No of complicated crown fractured teeth (*N* = 190)	1	n (%)	160 (84.2%)
	2	n (%)	22 (11.6%)
	3	n (%)	1 (0.5%)
	4	n (%)	6 (3.2%)
	7	n (%)	1 (0.5%)
	Total	N	238
Crown-root fracture (*N* = 1291)	No	n (%)	1274 (98.7%)
	Yes	n (%)	17 (1.3%)
Root fracture (*N* = 1291)	No	n (%)	1266 (98.1%)
	Yes	n (%)	25 (1.9%)
No of teeth with root fracture (*N* = 25)	1	n (%)	19 (76.0%)
	2	n (%)	6 (24.0%)
	Total	N	31
Enamel infraction (*N* = 1291)	No	n (%)	1289 (99.9%)
	Yes	n (%)	2 (0.2%)
Jaw fracture (*N* = 1291)	No	n (%)	1286 (99.6%)
	Yes	n (%)	5 (0.4%)

*IQR* interquartile range, *no* number

**Table 2:** Differences in TDI cases and injury data according to the traumatized tooth (column percentages)

Variable	Metric	Deciduous	Permanent	*P*
Gender (*N* = 1334)	Female - n (%)	494 (40.7%)	44 (36.4%)	0.35^$^
	Male - n (%)	719 (59.3%)	77 (63.6%)	
Dental phase (*N* = 1334)	Deciduous	924 (76.2%)	0 (0%)	< 0.001*
	Mixed	289 (23.8%)	66 (54.6%)	
	Permanent	0 (0%)	55 (45.5%)	
Age (years) (*N* = 1334)	Median (IQR) [range]	2.8 (1.7, 5.8) [0.1, 14.3]	10.0 (7.2, 13.4) [5.4, 16.2]	< 0.001^‡^
Age-group (*N* = 1334)	0–6.0 years - n (%)	920 (75.9%)	5 (4.1%)	< 0.001^$^
	6.1–12.0 years - n (%)	281 (23.2%)	63 (52.1%)	
	12.1–18.0 years - n (%)	12 (1.0%)	53 (43.8%)	
Accident year (*N* = 1334)	2010 - n (%)	151 (12.5%)	16 (13.2%)	0.05^$^
	2011 - n (%)	178 (14.7%)	14 (11.6%)	
	2012 - n (%)	120 (9.9%)	4 (3.3%)	
	2013 - n (%)	113 (9.3%)	7 (5.8%)	
	2014 - n (%)	101 (8.3%)	16 (13.2%)	
	2015 - n (%)	151 (12.5%)	23 (19.0%)	
	2016 - n (%)	123 (10.1%)	10 (8.3%)	
	2017 - n (%)	106 (8.7%)	15 (12.4%)	
	2018 - n (%)	103 (8.5%)	8 (6.6%)	
	2019 - n (%)	67 (5.5%)	8 (6.6%)	
Accident month (*N* = 1334)	January - n (%)	92 (7.6%)	8 (6.6%)	0.73^$^
	February - n (%)	88 (7.4%)	7 (5.8%)	
	March - n (%)	100 (8.2%)	13 (10.7%)	
	April - n (%)	120 (9.9%)	14 (11.6%)	
	May - n (%)	112 (9.2%)	11 (9.1%)	
	June - n (%)	101 (8.3%)	14 (11.6%)	
	July - n (%)	102 (8.4%)	6 (5.0%)	
	August - n (%)	90 (7.4%)	7 (5.8%)	
	September - n (%)	85 (7.0%)	8 (6.6%)	
	October - n (%)	122 (10.1%)	13 (10.7%)	
	November - n (%)	99 (8.2%)	12 (9.9%)	
	December - n (%)	102 (8.4%)	5 (4.1%)	
Accident day (*N* = 1334)	Monday - n (%)	186 (15.3%)	19 (15.7%)	0.34^$^
	Tuesday - n (%)	185 (15.3%)	19 (15.7%)	
	Wednesday - n (%)	186 (15.3%)	18 (14.9%)	
	Thursday - n (%)	196 (16.2%)	24 (19.8%)	
	Friday - n (%)	171 (14.1%)	23 (19.0%)	
	Saturday - n (%)	139 (11.5%)	10 (8.3%)	
	Sunday - n (%)	150 (12.4%)	8 (6.6%)	
Time > 24 h (*N* = 1301)	No - n (%)	691 (58.4%)	91 (77.1%)	< 0.001*
	Yes - n (%)	492 (41.6%)	27 (22.9%)	
Accident place (*N* = 1167)	At home - n (%)	502 (47.5%)	18 (16.4%)	< 0.001*
	Outdoor - n (%)	301 (28.5%)	58 (52.7%)	
	Playground - n (%)	54 (5.1%)	2 (1.8%)	
	Swimming pool - n (%)	37 (3.5%)	11 (10.0%)	
	Preschool - n (%)	85 (8.0%)	0 (0%)	
	School - n (%)	78 (7.4%)	21 (19.1%)	
Accident cause (*N* = 1322)	Accident - n (%)	72 (6.0%)	21 (17.8%)	< 0.001^$^
	Bicycle - n (%)	89 (7.4%)	15 (12.7%)	
	Fall - n (%)	457 (38.0%)	23 (23.7%)	
	Fall of stairs - n (%)	72 (6.0%)	8 (6.8%)	
	Playtime - n (%)	365 (30.3%)	20 (17.0%)	
	Scooter - n (%)	56 (4.7%)	14 (11.9%)	
	Sport - n (%)	93 (7.7%)	12 (10.2%)	
Soft tissue trauma extraoral (*N* = 1334)	No - n (%)	755 (62.2%)	60 (49.6%)	0.006^$^
	Yes - n (%)	458 (37.8%)	61 (50.4%)	
Soft tissue trauma intraoral (*N* = 1334)	No - n (%)	670 (55.2%)	59 (48.8%)	0.17^$^
	Yes - n (%)	543 (44.8%)	62 (51.2%)	
Injury type (*N* = 1334)	Injuries to the periodontal tissues - n (%)	629 (51.9%)	45 (37.2%)	< 0.001*
	Injuries to the hard dental tissues and the pulp - n (%)	327 (27.0%)	28 (23.1%)	
	Combined injury (injuries to the periodontal tissues & injuries to the hard dental tissues and the pulp) - n (%)	251 (20.7%)	47 (38.8%)	
	Soft-tissue injury - n (%)	6 (0.5%)	1 (0.8%)	
Avulsion (*N* = 1334)	No - n (%)	1090 (91.7%)	99 (81.8%)	0.007^$^
	Yes - n (%)	123 (10.1%)	22 (18.2%)	
No of avulsed teeth (*N* = 145)	1 - n (%)	95 (77.2%)	17 (77.3%)	1.00^$^
	> 1 - n (%)	28 (22.8%)	5 (22.7%)	
Lateral luxation (*N* = 1334)	No - n (%)	918 (75.7%)	85 (70.3%)	0.19^$^
	Yes - n (%)	295 (24.3%)	36 (29.8%)	
No of lateral luxated teeth (*N* = 331)	1 - n (%)	156 (52.9%)	16 (44.4%)	0.34^$^
	> 1 - n (%)	139 (47.1%)	20 (55.6%)	
Subluxation (*N* = 1334)	No - n (%)	949 (78.2%)	90 (74.4%)	0.33^$^
	Yes - n (%)	264 (21.8%)	31 (25.6%)	
No of subluxated teeth (*N* = 295)	1 - n (%)	103 (39.0%)	9 (29.0%)	0.28^$^
	> 1 - n (%)	161 (61.0%)	22 (71.0%)	
Concussion (*N* = 1334)	No - n (%)	321 (26.5%)	25 (20.7%)	0.17^$^
	Yes - n (%)	892 (73.5%)	96 (79.3%)	
No of concussed teeth (*N* = 988)	Median (IQR)	2.0 (2.0, 5.0)	4.0 (2.0, 6.0)	0.01^‡^
Intrusive luxation (*N* = 1334)	No - n (%)	1047 (86.3%)	113 (93.4%)	0.02^$^
	Yes - n (%)	166 (13.7%)	8 (6.6%)	
No of intrusive luxated teeth (*N* = 174)	1 - n (%)	92 (55.4%)	7 (87.5%)	0.14*
	> 1 - n (%)	74 (44.6%)	1 (12.5%)	
Extrusive luxation (*N* = 1334)	No - n (%)	1173 (96.7%)	116 (95.9%)	0.63^$^
	Yes - n (%)	40 (3.3%)	5 (4.1%)	
No of extrusive luxated teeth (*N* = 45)	1 - n (%)	29 (72.5%)	3 (60.0%)	0.62*
	> 1 - n (%)	11 (27.5%)	2 (40.0%)	
Enamel fracture (*N* = 1334)	No - n (%)	963 (79.4%)	100 (82.6%)	0.40^$^
	Yes - n (%)	250 (20.6%)	21 (17.4%)	
No of enamel fractured teeth (*N* = 271)	1 - n (%)	163 (65.2%)	11 (52.4%)	0.24^$^
	> 1 - n (%)	87 (34.8%)	10 (47.6%)	
Uncomplicated crown fracture (*N* = 1334)	No - n (%)	1006 (82.9%)	77 (63.6%)	< 0.001^$^
	Yes - n (%)	207 (17.1%)	44 (36.4%)	
No of uncomplicated crown fractured teeth (*N* = 251)	1 - n (%)	153 (73.9%)	33 (75.0%)	0.88^$^
	> 1 - n (%)	54 (26.1%)	11 (25.0%)	
Complicated crown fracture (*N* = 1334)	No - n (%)	1039 (85.7%)	101 (83.5%)	0.52^$^
	Yes - n (%)	174 (14.3%)	20 (16.5%)	
No of complicated crown fractured teeth (*N* = 194)	1 - n (%)	148 (85.1%)	15 (75.0%)	0.33*
	> 1 - n (%)	26 (14.9%)	5 (25.0%)	
Crown-root fracture (*N* = 1334)	No - n (%)	1197 (98.7%)	120 (99.2%)	1.00*
	Yes - n (%)	16 (1.3%)	1 (0.8%)	
Root fracture (*N* = 1334)	No - n (%)	1201 (99.0%)	108 (89.3%)	< 0.001*
	Yes - n (%)	12 (1.0%)	13 (10.7%)	
No of teeth with root fracture (*N* = 25)	1 - n (%)	9 (75.0%)	10 (76.9%)	1.00*
	> 1 - n (%)	3 (25.0%)	3 (23.1%)	
Enamel infraction (*N* = 1334)	No - n (%)	1211 (99.8%)	121 (100.0%)	1.00*
	Yes - n (%)	2 (0.2%)	0 (0%)	
Jaw fracture (*N* = 1334)	No - n (%)	1209 (99.7%)	120 (99.2%)	0.38*
	Yes - n (%)	4 (0.3%)	1 (0.8%)	

*IQR* interquartile range, *No* number

* from Fisher’s exact test

$ from chi-squared test

^‡^ from Kruskal–Wallis test

**Table 3:** Differences in injury characteristics according to patient sex (column percentages)

Variable	Category	Female	Male	*P* (sex)	*P* (Interaction sex x dentition)
Time < 24 h (*N* = 1301)	No - n (%)	306 (59.0%)	476 (60.9%)	0.49*	0.58
	Yes - n (%)	213 (41.0%)	306 (39.1%)		
Soft tissue trauma extraoral (*N* = 1334)	No - n (%)	315 (58.6%)	500 (62.8%)	0.12*	0.17
	Yes - n (%)	223 (41.5%)	296 (37.2%)		
Soft tissue trauma intraoral (*N* = 1334)	No - n (%)	299 (55.6%)	430 (54.0%)	0.58*	0.65
	Yes - n (%)	239 (44.4%)	366 (46.0%)		
Injury type (*N* = 1334)	Injuries to the periodontal tissues - n (%)	278 (51.7%)	396 (49.8%)	0.39^¥^	
	Injuries to the hard dental tissues and the pulp - n (%)	135 (25.1%)	220 (27.6%)		
	Combined injury (injuries to the periodontal tissues & injuries to the hard dental tissues and the pulp) - n (%)	124 (23.1%)	174 (21.9%)		
	Soft-tissue injury - n (%)	1 (0.2%)	6 (0.8%)		
Avulsion (*N* = 1334)	No - n (%)	480 (89.2%)	709 (89.1%)	0.93*	0.99
	Yes - n (%)	58 (10.8%)	87 (10.9%)		
Lateral Luxation (*N* = 1334)	No - n (%)	406 (75.5%)	597 (75.0%)	0.85*	0.19
	Yes - n (%)	132 (24.5%)	199 (25.0%)		
Subluxation (*N* = 1334)	No - n (%)	427 (79.4%)	612 (76.9%)	0.28*	0.83
	Yes - n (%)	111 (20.6%)	184 (23.1%)		
Concussion (*N* = 1334)	No - n (%)	134 (24.9%)	212 (26.6%)	0.48*	0.51
	Yes - n (%)	404 (75.1%)	584 (73.4%)		
Intrusive luxation (*N* = 1334)	No - n (%)	469 (87.2%)	691 (86.8%)	0.85*	0.90
	Yes - n (%)	69 (12.8%)	105 (13.2%)		
Extrusive luxation (*N* = 1334)	No - n (%)	514 (95.5%)	775 (97.4%)	0.07*	0.60
	Yes - n (%)	24 (4.5%)	21 (2.6%)		
Enamel fracture (*N* = 1334)	No - n (%)	428 (79.6%)	635 (79.8%)	0.92*	0.39
	Yes - n (%)	110 (20.5%)	161 (20.2%)		
Uncomplicated crown fracture (*N* = 1334)	No - n (%)	444 (82.5%)	639 (80.3%)	0.30*	0.63
	Yes - n (%)	94 (17.5%)	157 (19.7%)		
Complicated crown fracture (*N* = 1334)	No - n (%)	457 (84.9%)	683 (85.8%)	0.66*	0.77
	Yes - n (%)	81 (15.1%)	113 (14.2%)		
Crown-root fracture (*N* = 1334)	No - n (%)	530 (98.5%)	787 (98.9%)	0.57*	NT
	Yes - n (%)	8 (1.5%)	9 (1.1%)		
Root fracture (*N* = 1334)	No - n (%)	528 (98.1%)	781 (98.1%)	0.97*	0.94
	Yes - n (%)	10 (1.9%)	15 (1.9%)		
Enamel infraction(*N* = 1334)	No - n (%)	537 (99.8%)	795 (99.9%)	0.78*	NT
	Yes - n (%)	1 (0.2%)	1 (0.1%)		
Jaw fracture (*N* = 1334)	No - n (%)	537 (99.8%)	792 (99.5%)	0.37*	NT
	Yes - n (%)	1 (0.2%)	4 (0.5%)		

*CI* confidence interval, *OR* odds ratio

* from logistic regression

^¥^ from Fisher’s exact test

**Table 4:** Place and cause of accident in relation to accident type (positive row percentages; fisher’s exact test)

a											
		Avulsion		Lateral luxation		Subluxation		Concussion		Intrusive Luxation	
Variable	Category	n (%)	P	n (%)	P	n (%)	P	n (%)	P	n (%)	P
Accident place (N=1167)	At home	43 (8.3%)	0.002	122 (23.5%)	0.19	120 (23.1%)	0.06	384 (70.8%)	0.73	82 (15.8%)	0.18
	Outdoor	52 (14.5%)		92 (25.6%)		81 (22.6%)		271 (75.4%)		36 (10.0%)	
	Playground	8 (14.3%)		14 (25.0%)		7 (12.5%)		40 (71.4%)		9 (16.1%)	
	Swimming pool	9 (18.8%)		20 (41.7%)		11 (22.9%)		36 (75.0%)		8 (16.7%)	
	Preschool	4 (4.7%)		22 (25.9%)		20 (23.5%)		64 (75.3%)		11 (12.9%)	
	School	17 (17.2%)		26 (26.3%)		34 (34.3%)		72 (72.7%)		11 (11.1%)	
Accident cause (N=1322)	Accident	11 (11.8%)	<0.001	23 (24.7%)	0.09	19 (20.4%)	0.09	77 (82.8%)	0.10	6 (6.5%)	0.22
	Bicycle	16 (15.4%)		33 (31.7%)		26 (25.0%)		78 (75.0%)		10 (9.6%)	
	Fall	36 (7.4%)		122 (25.2%)		88 (18.1%)		359 (74.0%)		69 (14.2%)	
	Fall of stairs	18 (22.5%)		25 (31.3%)		24 (30.0%)		49 (61.3%)		15 (18.8%)	
	Playtime	31 (8.1%)		87 (22.6%)		92 (23.9%)		287 (74.6%)		53 (13.8%)	
	Scooter	9 (12.9%)		10 (14.3%)		20 (28.6%)		53 (75.7%)		7 (10.0%)	
	Sport	20 (19.1%)		30 (28.6%)		24 (22.9%)		76 (72.4%)		13 (12.4%)	

b											
		Enamel fracture		Uncomplicated crown fracture		Complicated crown fracture					
Variable	Category	n (%)	P	n (%)	*P*	n (%)	P				
Accident place (N=1167)	At home	94 (18.1%)	0.57	79 (15.2%)	<0.001	72 (13.9%)	0.02				
	Outdoor	73 (20.3%)		82 (22.8%)		56 (15.6%)					
	Playground	15 (26.8%)		14 (25.0%)		12 (21.4%)					
	Swimming pool	10 (20.8%)		19 (39.6%)		6 (12.5%)					
	Preschool	20 (23.5%)		7 (8.2%)		6 (7.1%)					
	School	21 (21.2%)		18 (18.2%)		6 (6.1%)					
Accident cause (N=1322)	Accident	15 (16.1%)	0.30	22 (23.7%)	0.06	16 (17.2%)	0.45				
	Bicycle	27 (26.0%)		23 (22.1%)		17 (16.4%)					
	Fall	89 (18.4%)		85 (17.5%)		66 (13.6%)					
	Fall of stairs	17 (21.3%)		11 (13.8%)		6 (7.5%)					
	Playtime	78 (20.3%)		63 (16.4%)		57 (14.8%)					
	Scooter	12 (17.1%)		21 (30.0%)		12 (17.1%)					
	Sport	28 (26.7%)		23 (21.9%)		18 (17.1%)

**Table 5:** Place and cause of accident in relation to age-group (column percentages; fisher’s exact test)

		0–6.0 years	6.1–12.0 years	12.1–18.0 years	
Variable	Category	*n* (%)	*n* (%)	*n* (%)	*P*
Accident place (*N* = 1127)	At home	451 (57.5%)	53 (18.7%)	10 (17.2%)	NC
	Outdoor	187 (23.8%)	124 (43.7%)	30 (51.7%)	
	Playground	46 (5.9%)	9 (3.2%)	1 (1.7%)	
	Swimming pool	9 (1.2%)	28 (9.9%)	5 (8.6%)	
	Preschool	83 (10.6%)	2 (0.7%)	0 (0%)	
	School	9 (1.2%)	68 (23.9%)	12 (20.7%)	
Accident cause (*N* = 1280)	Accident	41 (4.5%)	33 (10.8%)	9 (14.3%)	NC
	Bicycle	59 (6.5%)	30 (9.8%)	12 (19.1%)	
	Fall	392 (43.0%)	68 (22.3%)	16 (25.4%)	
	Fall of stairs	59 (6.5%)	13 (4.3%)	2 (3.2%)	
	Playtime	289 (31.7%)	79 (25.9%)	9 (14.3%)	
	Scooter	17 (1.9%)	45 (14.8%)	5 (7.9%)	
	Sport	55 (6.0%)	37 (12.1%)	10 (15.9%)	

NC, *p* value for Fisher’s exact test not calculable

**Table 6:** Time to come for emergency treatment according to weekday (row percentages; chi-squared test)

Accident day	Time < 24 h *n* (%)	Time > 24 h *n* (%)	*P*
Monday	136 (67.3%)	66 (32.7%)	< 0.001
Tuesday	138 (69.0%)	62 (31.0%)	
Wednesday	142 (70.7%)	59 (29.4%)	
Thursday	142 (67.9%)	67 (32.0%)	
Friday	114 (60.0%)	76 (40.0%)	
Saturday	43 (29.9%)	101 (70.1%)	
Sunday	67 (43.2%)	88 (56.8%)

**Table 7:** Time to come for emergency treatment according to traumatic dental injury (row percentages)

Variable	Category	Time < 24 h *n* (%)	Time > 24 h *n* (%)	*P*
Injury type (*N* = 1301)	Injuries to the periodontal tissues - n (%)	444 (67.5%)	214 (32.5%)	< 0.001*
	Injuries to the hard dental tissues and the pulp - n (%)	145 (42.2%)	199 (57.9%)	
	Combined injury (injuries to the periodontal tissues & injuries to the hard dental tissues and the pulp) - n (%)	188 (64.4%)	104 (35.6%)	
	Soft-tissue injury - n (%)	5 (71.4%)	2 (28.6%)	
Avulsion (*N* = 1301)	No - n (%)	685 (59.2%)	473 (40.9%)	0.04^†^
	Yes - n (%)	97 (67.8%)	46 (32.2%)	
Lateral luxation (*N* = 1301)	No - n (%)	548 (56.1%)	429 (43.9%)	< 0.001^†^
	Yes - n (%)	234 (72.2%)	90 (27.8%)	
Subluxation (*N* = 1301)	No - n (%)	597 (59.1%)	414 (41.0%)	0.14^†^
	Yes - n (%)	185 (63.8%)	105 (36.2%)	
Concussion (*N* = 1301)	No - n (%)	157 (46.7%)	179 (53.3%)	< 0.001^†^
	Yes - n (%)	625 (64.8%)	340 (35.2%)	
Intrusive luxation (*N* = 1301)	No - n (%)	660 (58.4%)	471 (41.6%)	0.001^†^
	Yes - n (%)	122 (71.8%)	48 (28.2%)	
Extrusive luxation (*N* = 1301)	No - n (%)	742 (59.0%)	515 (41.0%)	< 0.001*
	Yes - n (%)	40 (90.9%)	4 (9.1%)	
Enamel fracture (*N* = 1301)	No - n (%)	633 (61.2%)	402 (38.8%)	0.12^†^
	Yes - n (%)	149 (56.0%)	117 (44.0%)	
Uncomplicated crown fracture (*N* = 1301)	No - n (%)	643 (60.9%)	413 (39.1%)	0.23^†^
	Yes - n (%)	139 (56.7%)	106 (43.3%)	
Complicated crown fracture (*N* = 1301)	No - n (%)	698 (62.8%)	414 (37.2%)	< 0.001^†^
	Yes - n (%)	84 (44.4%)	105 (55.6%)	
Crown-root fracture (*N* = 1301)	No - n (%)	776 (60.4%)	509 (39.6%)	0.06^†^
	Yes - n (%)	6 (37.5%)	10 (62.5%)	
Root fracture (*N* = 1301)	No - n (%)	767 (60.1%)	510 (39.9%)	0.81^†^
	Yes - n (%)	15 (62.5%)	9 (37.5%)	
Enamel infraction (*N* = 1301)	No - n (%)	781 (60.1%)	518 (39.9%)	1.00*
	Yes - n (%)	1 (50.0%)	1 (50.0%)	
Jaw fracture (*N* = 1301)	No - n (%)	778 (60.0%)	518 (40.0%)	0.65*
	Yes - n (%)	4 (80.0%)	1 (20.0%)	

* from Fisher’s exact test

^†^ from chi-squared test

**Table 8:** Traumatic dental injuries among cases having only single or multiple injuries (column percentages)

Variable	Single injury (*N* = 198)	Multiple injuries (*N* = 1086)	*P*
Avulsion - *n* (%)	28 (14.1%)	104 (9.6%)	0.05^†^
Lateral luxation - *n* (%)	61 (30.8%)	253 (23.3%)	0.02^†^
Subluxation - *n* (%)	44 (22.2%)	239 (22.0%)	0.95^†^
Concussion - *n* (%)	14 (7.1%)	935 (86.1%)	< 0.001^†^
Intrusive luxation - *n* (%)	33 (16.7%)	138 (12.7%)	0.13^†^
Extrusive luxation - *n* (%)	6 (3.0%)	36 (3.3%)	0.84^†^
Enamel fracture - *n* (%)	3 (1.5%)	260 (23.9%)	< 0.001*
Uncomplicated crown fracture - *n* (%)	4 (2.0%)	236 (21.7%)	< 0.001*
Complicated crown fracture - *n* (%)	2 (1.0%)	188 (17.3%)	< 0.001*
Crown-root fracture - *n* (%)	2 (1.0%)	15 (1.4%)	1.00*
Root fracture - *n* (%)	0 (0%)	25 (2.3%)	0.02*
Enamel infraction - *n* (%)	0 (0%)	2 (0.2%)	1.00*
Jaw fracture - *n* (%)	1 (0.5%)	4 (0.4%)	0.57*

* from Fisher’s exact test

^†^ from chi-squared test

**Table 9:** Traumatic dental injuries among cases having only single or multiple injuries (row percentages; chi-squared test)

Variable	Category	Injuries to the hard dental tissues and the pulp	Injuries to the periodontal tissues	Combined injury (injuries to the periodontal tissues & injuries to the hard dental tissues and the pulp)	*P*
Sex	Female - n (%)	132 (30.3%)	188 (43.1%)	116 (26.6%)	0.89
	Male - n (%)	213 (31.5%)	290 (42.9%)	173 (25.6%)	
Age group	0–6.0 years - n (%)	238 (30.6%)	366 (47.0%)	175 (22.5%)	< 0.001
	6.1–12.0 years - n (%)	78 (28.9%)	99 (36.7%)	93 (34.4%)	
	12.1–18.0 years - n (%)	29 (46.0%)	13 (20.6%)	21 (33.3%)	
Time < 24 h	No - n (%)	140 (21.5%)	332 (51.1%)	178 (27.4%)	< 0.001
	Yes - n (%)	194 (44.7%)	135 (31.1%)	105 (24.2%)

**Table 10:** Differences among patients having multiple injury instances and patient characteristics at each injury instance (column percentages)

		One injury/patient	Two injuries/patient	Three injuries/patient	Four injuries/patient	*P*
Total (*N* = 1236)	n (%)	1187 (96.0%)	44 (3.6%)	4 (0.3%)	1 (0.1%)	
		**One injury/patient**	**Multiple injuries/patient**			
Total (*N* = 1236)	n (%)	1187 (96.0%)	49 (4.0%)			
Age-group (*N* = 1236)	0–6.0 years - n (%)	847 (71.4%)	36 (73.5%)			1.00*
	6.1–12.0 years - n (%)	281 (23.7%)	11 (22.5%)			
	12.1–18.0 years - n (%)	59 (5.0%)	2 (4.1%)			
Sex	Female - n (%)	480 (40.4%)	18 (36.7%)			0.60^†^
	Male - n (%)	707 (59.6%)	31 (63.3%)			
		** 1 st injury** (*N* = 1236)	**2nd injury** (*N* = 49)	**3rd injury** (*N* = 5)	**4th injury** (*N* = 1)	
Age	Median (IQR)	2.9 (1.7, 7.1)	3.5 (2.5, 8.1)	5.7 (4.9, 7.8)	7.0 (-)	
Sex	Female - n (%)	498 (40.3%)	18 (36.7%)	3 (60.0%)	0 (0%)	0.69*
	Male - n (%)	738 (59.7%)	31 (63.3%)	2 (40.0%)	1 (100.0%)	

*IQR* interquartile range

* from Fisher’s exact test

^†^ from chi-squared test

## TDI characteristics and frequency

Extraoral/intraoral soft-tissue injuries were seen in 38.3% and 44.6% of the cases, respectively (Table 1). TDIs were mostly categorized as injuries to the periodontal tissues (50.4%), followed by injuries to the hard dental tissues and the pulp (27.1%), combined injury (i.e., injuries to the periodontal tissues and injuries to the hard dental tissues and the pulp; 21.9%), and soft-tissue injury alone (0.5%). Tooth avulsion was seen in 10.2% of the cases, with usually one (77.3%) or two (15.2%) teeth affected. Lateral luxation was seen in 24.3% of the cases, with one (52.2%) or two (35.4%) teeth usually affected. Subluxation was seen in 21.9% of the cases, with one or two teeth usually affected (38.9% and 42.8%, respectively). Concussion, which was defined as pain on percussion/palpation without any signs of sulcus bleeding, was seen very often (73.5% of the cases), with a median of two teeth (IQR 2.0–5.0 teeth) per patient affected. Intrusive luxation was seen in 13.3% of the cases, with one or two teeth intruded (56.7% and 36.3%, respectively). Extrusive luxation was seen less often (3.3%) and mostly pertained to single teeth (73.8%). Regarding injuries to the hard dental tissues and the pulp, fractures of enamel only were found most often (20.4%), followed by uncomplicated crown fracture (18.6%), complicated crown fracture (14.7%), root fractures (1.9%), crown-root fractures (1.3%), and enamel infraction (0.2%).

## Comparative analysis of TDIs in deciduous and permanent teeth

**Fig. 1:**
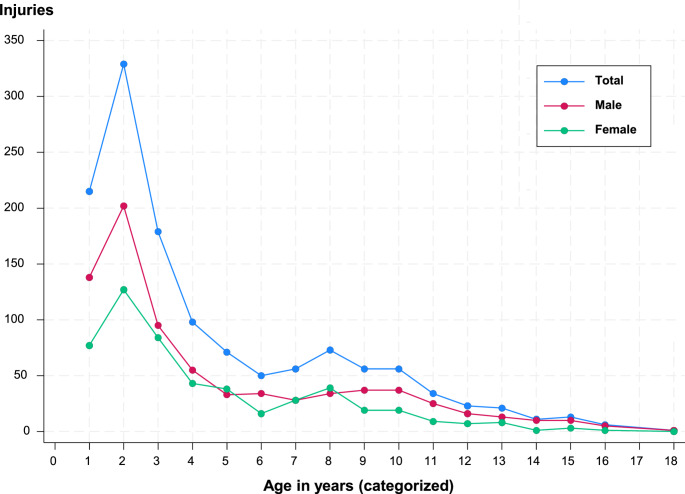
Traumatic dental injuries between 2010–2019 in our study

No significant differences were seen in terms of accidents per year (Fig. 1) and accidents per month or Day. Patients visited the dentist within 24 h after the dental trauma more often when a permanent tooth was injured than when a deciduous tooth was injured (77.1% versus 58.4%, respectively; *p* < 0.001; Table 2). Deciduous teeth were primarily injured at home (47.5%) or outdoors (28.5%), while permanent teeth were injured mostly outdoors (52.7%) or at school (19.1%; *p* < 0.001; Table 2). Deciduous teeth were injured mostly from falls (38.0%) or during playtime (30.3%), while permanent teeth were injured during a variety of activities (i.e., falls [23.4%], accidents [17.8%], and playtime [17.0%]). Extraoral soft-tissue injuries were associated more with the trauma of permanent teeth than deciduous teeth (50.4% and 37.8%, respectively; *p* = 0.006; Table 2). Both primary and permanent dentitions exhibited more injuries to the periodontal tissues (51.9% and 37.2%, respectively) than injuries to the hard dental tissues and the pulp (27.0% and 23.1%, respectively; *p* < 0.001; Table 2). Compared to deciduous teeth, permanent teeth were more often affected by avulsion (18.2% versus 10.1%), concussion (median affected: 4.0 versus 2.0 teeth), lateral luxation (29.8% versus 24.3%), uncomplicated crown fracture (36.4% versus 17.1%), and root fracture (10.7% versus 1.0%). In contrast, deciduous teeth showed more intrusive luxation than permanent teeth (13.7% versus 6.6%; Table 2).

**Fig. 2:**
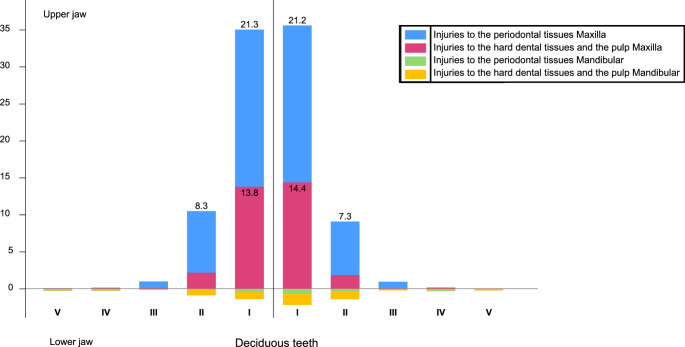
Number and type of deciduous teeth affected by trauma in the present study

**Fig. 3:**
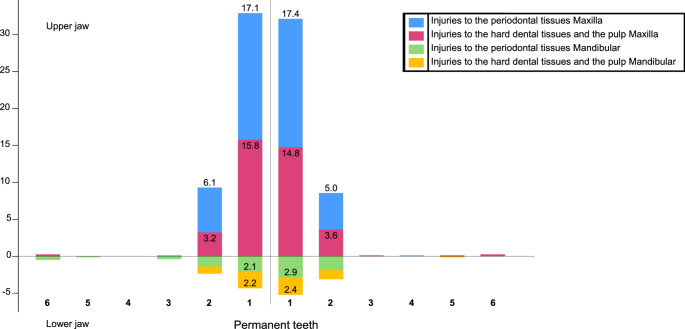
Number and type of permanent teeth affected by trauma in the present study

Appendix 1 shows which teeth were most affected by each traumatic injury type, stratified by dentition. In all instances, the single most affected tooth was the upper central incisor (left or right), both in the deciduous (Fig. 2) and permanent dentitions (Fig. 3). There was a strong tendency in the maxilla (in both dentitions) for avulsion, lateral luxation, subluxation, intrusive luxation, extrusive luxation, enamel fracture, uncomplicated crown fracture, complicated crown fracture, crown-root fracture, and root fracture. The only exception was concussion, which was observed at similar rates in the maxilla and the mandible, in both deciduous (63.9% and 36.1%, respectively) and permanent dentitions (52.7% and 47.3%, respectively). Usually, the central incisor was the tooth most affected by far, with the lateral incisors second and only a few canines or posterior teeth being affected. This outcome was true for the same group of TDIs as before (i.e., avulsion, lateral luxation, subluxation, intrusive luxation, extrusive luxation, enamel fracture, uncomplicated crown fracture, complicated crown fracture, crown-root fracture, and root fracture). Again, the only exception was concussion, where central and lateral incisors were equally affected. These two teeth accounted for 80–90% of the cases. Posterior teeth were seldom affected in both the primary and permanent dentitions. The most common injuries were avulsion (3.6% and 0.0%, respectively), concussion (4.0% and 8.5%, respectively), extrusive luxation (0.0% and 7.7%, respectively), enamel fracture (0.4% and 3.1%, respectively), uncomplicated crown fracture (7.9% and 2.2%, respectively), and complicated crown fracture (5.5% and 3.6%, respectively; Appendix 1).

### Sex-related differences in TDI occurrence and patterns

No significant differences in any aspect of TDIs were seen according to patient sex (Table 3). Similarly, no significant interaction in terms of sex with dentitions was found, which indicated that similar TDI patterns were seen among male and female patients for deciduous and permanent teeth.

### TDI types related to location and accident causation

Significant differences in accident locations and causes were seen for some TDI types (Tables 4a–b). Tooth avulsion predominantly happened at the swimming pool (18.8%), followed by at school (17.2%), outdoors (14.5%), and on playgrounds (14.3%) as opposed to other places (*p* = 0.002). The most common causes of avulsion were falling downstairs (22.5%), during sports (19.1%), and bicycle accidents (15.4%; *p* < 0.001; Table 4a). Uncomplicated crown fracture occurred more often at the swimming pool (39.6%), followed by the playground (25.0%) or outdoors (22.8%) than at other places (*p* < 0.001). Finally, complicated crown fractures happened more often on the playground (21.4%), outdoors (15.6%), or at home (13.9%) than at other places (*p* = 0.02; Table 4b).

### Age-related differences

Considerable differences were seen in the accident locations and causes among the various age groups (Table 5), even though these could not be tested appropriately due to data dilution in many small groups. In those 0–6.0 years old, most injuries happened at home (57.5%), while for those 6.1–12.0 and 12.1–18.0 years old, most injuries happened outdoors (43.7% and 51.7%, respectively). The major cause of injuries for those 0–6.0 years old was falls (43.0%), followed by playtime (31.7%). For those 6.1–12.0 years old, the major causes were playtime (25.9%) and falls (22.3%), while for those 12.1–18.0 years old, the major causes were falls (25.4%), bicycle accidents (19.1%), and sports activities (15.9%).

### Time from the accident to the dentist visit

Considerably different amounts of time elapsed from the TDI until patients visited the dentist (or were brought by their parents), with < 24 h elapsing in 59.2% of the cases (Table 1). Most TDI cases presented within 24 h on weekdays, with the highest rates on Wednesdays (70.7%), Tuesdays (69.0%), and Thursdays (67.9%), followed by Mondays (67.3%) and Fridays (60.0%). In contrast, lower rates were observed on weekends, with 43.2% on Sundays and 29.9% (*p* < 0.001) on Saturdays (Table 6). Considerable variations in the time before visiting the dentist were also seen according to injury type (Table 7). Patients visited the dentist within 24 h of the accident, most often for soft-tissue injuries (71.4%), followed by injuries to the periodontal tissues (67.5%), combined injuries (64.4%), and injuries to the hard dental tissues and the pulp alone (42.2%; *p* < 0.001). Concerning specific injuries, patients visited the dentist within 24 h of the accident more often when they had extrusive luxation (90.9%), lateral luxation (72.2%),intrusive luxation (71.8%), avulsion (67.8%), or concussion (64.8%; *p* < 0.05 in all instances). However, fractures (either to the enamel solely or the enamel-dentin) did not result in timely dentist visits, while uncomplicated crown fractures were associated with delayed presentation (44.4%; *p* < 0.001).

### Accuracy of data extraction procedure

A random sample of 200 patient files was independently checked by a second investigator to verify that the accident place, accident cause, and TDI type had been accurately extracted. The data extraction showed a high degree of accuracy and had a concordance correlation coefficient of 0.96 (95% CI 0.95 to 0.97).

##  Discussion

This study retrospectively analyzed a large sample of children with TDIs who were treated in the Clinic of Orthodontics and Pediatric Dentistry of the Center of Dental Medicine, University of Zurich from 2010 to 2019. The results indicate that many major and minor traumatic injuries occurred, and trends were observed that could have consequences for the adequate management of TDIs.

Temporal variations were seen in the incidence of TDIs across the months of the year and the days of the week. The reporting of TDIs showed two peaks: one in October (10.3% of the sample) and one in April (9.9% of the sample). This trend might be due to local public school holidays, which could lead to more outdoor activities. Some previous studies have indicated September-October and April-May as the months with higher reported incidences of TDIs among children [31–33], which is consistent with our findings. However, other studies have reported peaks during the warmer months, such as June and July, possibly due to increased time spent outdoors and greater physical activity during summer breaks (holiday breaks) [34, 35]. For example, a study by Gfeller et al. [36] conducted at the University of Bern in Switzerland found the highest incidence of TDIs to be in March and July. While both their study and this current study were conducted in Switzerland, direct comparison is challenging due to differences in the study populations. In contrast to Gfeller et al. [36], who included patients ranging in age from 10 months to 91 years, this study focused exclusively on patients aged 0 to 18 years. These variations in age distribution may have influenced the temporal patterns found for TDIs, as risk factors and activity levels differ significantly across age groups. Nevertheless, the comparison is still useful in showing how demographics and regional factors can influence the occurrence of TDIs and emphasizing the necessity of targeted preventive strategies. Furthermore, discrepancies between studies might be explained by differences in public holidays, which vary between different countries. This variation could potentially influence children’s activity patterns and injury risks.

In terms of weekday distribution, this study reported that TDIs increased over the first days of the week, peaked on Thursdays, and reached their lowest point on weekends. This finding aligns with some previous data [37, 38] but diverges from studies that reported increased trauma rates during the weekend [31, 39, 40]. Gfeller et al. [36] identified Tuesday as the Day with the lowest TDI incidence and Friday as the peak Day, followed by Saturday. In contrast, as mentioned above, the current study found the lowest incidence on weekends. A possible explanation for the relatively low rates recorded during weekends in this study is the closure of the university clinic these days. Consequently, many patients may have sought care at hospitals or emergency dental facilities instead. It is also important to note that this study only involved patients aged 0 to 18 and was carried out at a pediatric dental department. On the other hand, the study by Gfeller et al. [36] was based in an oral surgery and stomatology department, which treats patients across all age groups, including adults. These differences in clinical settings and patient demographics may account for some of the variation in the temporal distribution of TDI between the studies.

According to the results, TDIs for those 0 to 6.0 years old peaked at approximately 1.6 years, while those 6.1–12.0 years old peaked at 8.4 years, and those 12.1–18.0 years old peaked at 12.8 years (Table 1). These results align with Lam et al. [41], who found that patients 0.0–4.0 years old had the highest TDI frequency, followed by those aged 5.0–9.0 and 10.0–14.0 years old. One explanation for elevated TDIs in children aged 0 to 6.0 years, especially children under 3.0, could be the unfinished development of their walking and motor coordination, which could make them more likely to incur accidents. In contrast, Wright et al. [42] showed that 43% of TDIs occurred in the 8.0–11.0-year-old age group, while Eyuboglu et al. [43] showed that TDIs peaked at age 10.0. Variations in age group classifications, sampling methods, and inclusion criteria can explain these differences. Nonetheless, the literature shows a clear trend indicating that very young children and those of late school age are more prone to TDIs, which matches the age distribution found in the current study. Another possible explanation for the observed peak in TDIs between 0 and 6.0 years old is that many parents may initially present their children with TDIs to children’s hospitals rather than dental clinics. In such cases, hospitals may refer these young patients to university dental care, as many of them have not yet had prior contact with a dentist. In contrast, parents may generally be more inclined to seek immediate care from their private dentist for dental trauma in older children.

This present study found that the time that elapsed from the accident’s occurrence until the child visited the dentist varied considerably. In over half of the cases (59.2%), the delay was up to 24 h (Table 1). Other studies have reported that sometimes, patients wait for days, months, or even years after the initial injury and visit a dentist only after complications have arisen [44, 45]. In this study, the proportion of children visiting the dentist within 24 h of a dental injury was relatively consistent throughout the week (60.0–70.7%) but considerably lower during the weekend (29.9–43.2%). This finding further reinforces the previously reported notion that dental injuries occurring during the weekend are only reported the following week. Sometimes, patients experiencing TDIs on the weekend may seek treatment at other emergency dental practices or hospitals. Moreover, patients visited the dentist more often within the first 24 h when they had a concussion, lateral luxation, intrusive luxation, or extrusive luxation. This finding is important because the time that has elapsed between the occurrence of a TDI and a dentist visit is crucial for the successful prognosis and management of injured teeth. The International Association of Dental Traumatology guidelines clearly state that in instances of injuries to the periodontal tissues or the hard dental tissues and pulp, treatment should commence as soon as possible [24]. The same is suggested in pertinent guidelines for avulsed teeth [26]. Minimizing the time the tooth is left out of the mouth (and especially not in a storage medium) is crucial to the survival of the periodontal ligament cells, which become inviable after an extra-alveolar dry time of 30 to 60 min [46, 47]. Another explanation for the delayed presentation, often beyond 24 h, of patients with severe dental injuries may be that they initially seek medical treatment for other physical injuries at a hospital. After their first medical treatment, these individuals are then referred to a dentist for dental care.

Regarding gender distribution, the study’s sample of patients with TDIs was predominantly comprised of boys (59.8% versus 40.2%; Table 1), even though this was not confirmed statistically (Table 3) and conflicts with previous studies [6, 31, 35, 39]. Possible explanations for a higher rate of TDIs among boys include boys participating more often in high-intensity physical activity, having higher dopamine/epinephrine levels, or being more prone to violence than girls [31, 44, 48]. However, other studies have demonstrated that gender gaps are decreasing, since girls in Western culture have similar sports interests and are exposed to the same risk factors as boys [5, 49]. Gfeller et al. [36] also showed a higher prevalence of TDIs among boys; however, our results should be interpreted with caution because the observed gender difference was not statistically significant.

Furthermore, the results of the present study indicate that deciduous teeth sustain injuries predominantly at home (47.5%) and outdoors (28.5%), whereas permanent teeth are most frequently damaged outdoors (52.7%) and at school (19.1%). Dolic et al.’s [50] research indicated that the primary site of incidents involving deciduous teeth was the home (60.0%), whereas damage to permanent teeth predominantly occurred on the playground (59.4%). The leading causes of TDI in deciduous teeth (38.0%) and permanent teeth (23.7%) were falls, which has also been reported in other studies [51, 52]. Interestingly, playtime (17.0%), bicycle riding (12.7%), scooter use (11.9%), and sports (10.2%) were all associated with higher TDI frequencies in permanent teeth. Previous studies have shown that the frequencies of TDIs involving two-wheelers and sports increase among children aged 7 to 15 years [53, 54].

Furthermore, the results of the present study indicate that soft-tissue injuries external to the oral cavity are more commonly linked to trauma involving permanent teeth (50.4%) than deciduous teeth (37.8%; *p* = 0.006). According to Sae-Lim et al. [55], approximately half of all TDI patients have lesions on their oral soft tissues. Consequently, the use of protective gear (e.g., mouthguards and helmets) is crucial to potentially mitigating TDI severity.

In terms of injury type, injuries to the periodontal tissues were the most common for both deciduous teeth (51.9%) and permanent teeth (37.2%; *p* < 0.001). Regarding deciduous teeth, other studies have shown that injuries to the periodontal tissues occur more commonly than injuries to the hard dental tissues and pulp, which may be attributed to the elasticity of the alveolar bone and the lower crown-root ratio observed in these teeth [32, 56]. Other data indicate that injuries to the hard dental tissues and pulp are more common in permanent teeth [43, 56, 57]. The discrepancy between the results of other studies and the present study regarding permanent teeth may arise from two principal sources. First, the present investigation involved a smaller patient sample of permanent teeth. Second, it is plausible that children with tooth fractures may have sought care from private dentists, which potentially influenced the number of these patients presenting at the university clinic.

The most commonly affected tooth in both dentitions was the upper central incisor, either on the left or on the right. Canines and posterior teeth were infrequently affected. This finding aligns with other research indicating that most trauma cases concern the anterior teeth, primarily the maxillary central incisors [58–60]. Furthermore, a noticeable tendency toward the maxilla was found when the injury involved avulsion, lateral luxation, subluxation, intrusive luxation, extrusive luxation, enamel fracture, uncomplicated crown fracture, complicated crown fracture, crown-root fracture, and root fracture. Hence, a prophylactic retraction of protruded upper teeth [6] is a potentially meaningful measure.

Mahmoodi et al. [31] noted that 15.6% of patients suffered from avulsions, while avulsions accounted for 10.2% of the TDIs in this study. Furthermore, when types of injuries were compared, tooth avulsions showed statistically significant differences in the accident location and cause (*p* < 0.05). Avulsions occurred more commonly in swimming pools (18.8%), schools (17.2%), outdoor areas (14.5%), and playgrounds (14.3%), and at lower rates in the home (8.3%) and at preschool (4.7%). In terms of the causes of tooth avulsions, Gfeller et al. [36] reported that bicycle accidents had the highest probability of resulting in tooth avulsions. While our findings also identified bicycle accidents as a relevant cause, they accounted for only 15.4% of avulsion cases in this study and were not the leading cause. The leading causes of tooth avulsions in the present study were falling downstairs (22.5%), followed by sports activities (19.1%), and bicycle accidents (15.4%). This finding underscores the essential importance of adult oversight and protective measures in these situations and suggests that strategies focused on preventing injuries should prioritize stair safety, sports rules, and protective cycling equipment.

Knowledge and awareness of the potential impacts of accident-related TDIs, the role of preventive strategies, and the importance of appropriate and timely TDI management vary among the various parties broadly involved in TDIs. Less than half of the parents of patients with TDIs report being aware of the immediate actions that should be performed in the case of an avulsed tooth, and less than a quarter reported being aware of the appropriate storage medium for an avulsed tooth [61]. Similarly, less than half of these parents reported being confident in their ability to identify an injured tooth, clean an avulsed tooth, and replant an avulsed tooth in their child [61, 62]. Awareness among schoolteachers, athletes, and coaches paints a similarly bleak picture, even though teachers are often the first people to encounter a child’s TDI. Less than half of schoolteachers reported being able to differentiate between primary versus permanent dentition or being aware of the importance of replantation timing, and less than a fifth of schoolteachers had dental trauma training [63]. Athletic coaches and sports coaches were considerably less aware of these matters [9]. Non-dental healthcare professionals also seem to have low levels of awareness regarding TDIs, with only 30–40% of them reporting having received any dental trauma training or being aware of the appropriate storage and transfer medium for avulsed teeth and the importance of their timely replantation [64]. Awareness among dental professionals is considerably higher, with almost 80% of them reporting being aware of the importance of timely replantation of avulsed teeth and almost 70% being aware of the appropriate storage and transfer medium for avulsed teeth [65]. Although these awareness rates are higher than those of parents, schoolteachers, or sports coaches, they can still be improved. Thus, TDI awareness should be part of a contemporary dental school curriculum. The low TDI awareness rates among non-dental professionals may contribute to the underreporting or late reporting of TDIs and, eventually, to their late or even suboptimal management [62]. As such, it is crucial that awareness levels are raised among all potentially involved entities. Efforts from scientific societies, government agencies, and insurance companies should be put in place towards this aim.

### Limitations

This study has several limitations. First, the sample only includes patients presenting to a university pediatric dentistry clinic as a result of a TDI. The results do not depict the general population. Thus, the study does not offer an accurate measure of the incidence of TDIs and only reports on trends in dental injuries. Second, this retrospective observational study may have been more prone to bias than a prospective study. Third, much of the information included in this study was self-reported by the patients or their legal guardians/parents. Hence, the validity of the results depends on subjects’ memories and accurate reporting. Finally, the study is based on archival data on the included patients and does not include findings from radiographical images on a standard basis, which may have added more detail. Nevertheless, a potential strength of this study is the consistency in case evaluation and management, as all postgraduate students discussed their cases with the same program director and received standardized training under his supervision. This consistency may have reduced some of the typical weaknesses of retrospective studies by providing standardized methods for documenting and planning treatments, although it does not fully overcome the previously mentioned limitations.

## Conclusion

This study found that regarding children and adolescents exhibiting TDIs, significant differences were seen in injury mechanisms between deciduous and permanent teeth. Moreover, injuries to the periodontal tissues, with or without injuries to the hard dental tissues and the pulp, were the most common trauma found. Maxillary teeth were the most commonly injured teeth in both dentitions, while only a moderate percentage of children who sustained injuries visited the dentist within the first 24 h (59.2%). Raising awareness of these issues among parents, teachers, and dental professionals is crucial so that preventive TDI policies are enacted with appropriate protocols for their timely and effective management.

## Supplementary Information

Below is the link to the electronic supplementary material.

Supplementary Material 1

## Data Availability

The dataset for this study is openly available via Zenodo (https://doi.org/10.5281/zenodo.14047897).
