# Supplementary material for: Trends in accident-related traumatic dental injuries among children: a 10-year retrospective study of patients attending a university clinic
Source: Clin Oral Investig. 2025 Sep 15;29(10):450. doi: 10.1007/s00784-025-06546-4 (PMC12433922; doi:10.1007/s00784-025-06546-4)
Supplement: Supplementary file 1 — Supplementary Material 1 [file 784_2025_6546_MOESM1_ESM.pdf]

Trends in accident-related traumatic dental injuries among children: a 10-year retrospective study of patients attending a university clinic

Appendix

Apendix 1. Frequencies of affected teeth for each injury type.

	Deciduous			Permanent			Deciduous			Permanent			Deciduous			Permanent		
Trauma	Tooth #	n	%	Tooth #	n	%	Jaw	n	%	Jaw	n	%	Tooth	n	%	Tooth	n	%
Avulsion	61	36	32.1%	21	25	37.9%	Max	98	87.5%	Max	52	78.8%	I1	74	66.1%	I1	53	80.3%
	51	33	29.5%	11	18	27.3%	Mnd	14	12.5%	Mnd	14	21.2%	I2	29	25.9%	I2	13	19.7%
	52	12	10.7%	41	6	9.1%							C	5	4.5%	C	0	0.0%
	62	12	10.7%	22	5	7.6%							Other	4	3.6%	Other	0	0.0%
	71	4	3.6%	12	4	6.1%												
	72	3	2.7%	31	4	6.1%												
	63	2	1.8%	32	2	3.0%												
	82	2	1.8%	42	2	3.0%												
	83	2	1.8%	Total	66													
	55	1	0.9%															
	54	1	0.9%															
	53	1	0.9%															
	75	1	0.9%															
	81	1	0.9%															
	85	1	0.9%															
	Total	112																
Lateral Luxation	51	132	33.6%	11	46	39.3%	Max	369	93.9%	Max	105	89.7%	I1	271	69.0%	I1	91	77.8%
	61	125	31.8%	21	38	32.5%	Mnd	24	6.1%	Mnd	12	10.3%	I2	112	28.5%	I2	25	21.4%
	52	58	14.8%	12	14	12.0%							C	9	2.3%	C	0	0.0%
	62	46	11.7%	22	7	6.0%							Other	1	0.3%	Other	1	0.9%
	81	14	3.6%	31	4	3.4%												
	82	8	2.0%	41	3	2.6%												
	63	6	1.5%	32	2	1.7%												
	53	2	0.5%	42	2	1.7%												
	74	1	0.3%	45	1	0.9%												
	83	1	0.3%	Total	117													
	Total	393																
Subluxation	61	125	33.2%	11	56	29.8%	Max	347	92.3%	Max	160	85.1%	I1	256	68.1%	I1	128	68.1%
	51	115	30.6%	21	54	28.7%	Mnd	29	7.7%	Mnd	28	14.9%	I2	109	29.0%	I2	59	31.4%
	52	51	13.6%	12	26	13.8%							C	10	2.7%	C	1	0.5%
	62	46	12.2%	22	23	12.2%							Other	1	0.3%	Other	0	0.0%
	71	9	2.4%	31	9	4.8%												
	72	8	2.1%	41	9	4.8%												
	53	7	1.9%	42	6	3.2%												
	81	7	1.9%	32	4	2.1%												
	82	4	1.1%	13	1	0.5%												
	63	3	0.8%	Total	188													
	84	1	0.3%															

	Total	376																					
Concussion	61	389	16.7%		21	151	13.3%		Max	1492	63.9%		Max	596	52.7%		I1	1174	50.3%		I1	549	48.5%
	51	383	16.4%		11	144	12.7%		Mnd	843	36.1%		Mnd	536	47.3%		I2	976	41.8%		I2	458	40.5%
	62	313	13.4%		31	129	11.4%										C	92	3.9%		C	29	2.6%
	52	306	13.1%		41	125	11.0%										Other	93	4.0%		Other	96	8.5%
	81	206	8.8%		22	120	10.6%																
	71	196	8.4%		12	119	10.5%																
	82	179	7.7%		32	110	9.7%																
	72	178	7.6%		42	109	9.6%																
	63	32	1.4%		16	15	1.3%																
	53	26	1.1%		36	15	1.3%																
	73	17	0.7%		46	14	1.2%																
	83	17	0.7%		26	13	1.1%																
	64	16	0.7%		13	8	0.7%																
	75	15	0.6%		23	7	0.6%																
	65	13	0.6%		33	7	0.6%																
	85	13	0.6%		43	7	0.6%																
	74	12	0.5%		34	6	0.5%																
	54	11	0.5%		14	4	0.4%																
	84	10	0.4%		25	4	0.4%																
	55	3	0.1%		44	4	0.4%																
	Total	2335			15	3	0.3%																
					24	3	0.3%																
					27	3	0.3%																
					35	3	0.3%																
					45	3	0.3%																
					17	2	0.2%																
					37	2	0.2%																
					47	2	0.2%																
					Total	1132																	
Intrusive luxation	51	82	35.2%		21	17	41.5%		Max	225	96.6%		Max	39	95.1%		I1	169	72.5%		I1	34	82.9%
	61	82	35.2%		11	15	36.6%		Mnd	8	3.4%		Mnd	2	4.9%		I2	54	23.2%		I2	7	17.1%
	52	27	11.6%		12	5	12.2%										C	10	4.3%		C	0	0.0%
	62	24	10.3%		22	2	4.9%										Other	0	0.0%		Other	0	0.0%
	53	6	2.6%		31	1	2.4%																
	63	4	1.7%		41	1	2.4%																
	81	3	1.3%		Total	41																	
	82	3	1.3%																				
	71	2	0.9%																				
	Total	233																					
Extrusive Luxation	51	19	44.2%		21	6	46.2%		Max	35	81.4%		Max	13	100.0%		I1	35	81.4%		I1	9	69.2%
	61	12	27.9%		11	3	23.1%		Mnd	8	18.6%		Mnd	0	0.0%		I2	7	16.3%		I2	3	23.1%
	62	2	4.7%		22	3	23.1%										C	1	2.3%		C	0	0.0%
	71	2	4.7%		24	1	7.7%										Other	0	0.0%		Other	1	7.7%
	72	2	4.7%		Total	13																	

	81	2	4.7%																				
	82	2	4.7%																				
	52	1	2.3%																				
	63	1	2.3%																				
	Total	43																					
Enamel fracture	61	105	38.6%		11	32	25.2%		Max	251	92.3%		Max	86	67.7%		I1	220	80.9%		I1	83	65.4%
	51	102	37.5%		21	29	22.8%		Mnd	21	7.7%		Mnd	41	32.3%		I2	50	18.4%		I2	38	29.9%
	52	24	8.8%		22	13	10.2%										C	1	0.4%		C	2	1.6%
	62	18	6.6%		41	12	9.4%										Other	1	0.4%		Other	4	3.1%
	81	7	2.6%		31	10	7.9%																
	71	6	2.2%		42	10	7.9%																
	82	5	1.8%		12	8	6.3%																
	72	3	1.1%		32	7	5.5%																
	53	1	0.4%		16	1	0.8%																
	64	1	0.4%		23	1	0.8%																
	Total	272			25	1	0.8%																
					26	1	0.8%																
					35	1	0.8%																
					33	1	0.8%																
					Total	127																	
Uncomplicated crown fracture	51	60	39.7%		21	63	35.2%		Max	135	89.4%		Max	153	85.0%		I1	119	78.8%		I1	140	78.2%
	61	56	37.1%		11	62	34.6%		Mnd	16	10.6%		Mnd	27	15.0%		I2	16	10.6%		I2	33	18.4%
	52	8	5.3%		12	15	8.4%										C	4	2.6%		C	2	1.1%
	62	7	4.6%		22	12	6.7%										Other	12	7.9%		Other	4	2.2%
	84	4	2.6%		41	9	5.0%																
	74	3	2.0%		31	6	3.4%																
	81	3	2.0%		36	3	1.7%																
	73	2	1.3%		32	3	1.7%																
	55	1	0.7%		42	3	1.7%																
	54	1	0.7%		33	2	1.1%																
	63	1	0.7%		26	1	0.6%																
	64	1	0.7%		Total	179																	
	75	1	0.7%																				
	82	1	0.7%																				
	83	1	0.7%																				
	85	1	0.7%																				
	Total	151																					
Complicated crown fracture	61	85	46.7%		21	23	41.8%		Max	172	100.0%		Max	49	89.1%		I1	158	86.8%		I1	45	81.8%
	51	70	38.5%		11	20	36.4%		Mnd	0	0.0%		Mnd	6	10.9%		I2	14	7.7%		I2	8	14.5%
	52	7	3.8%		22	3	5.5%										C	0	0.0%		C	0	0.0%
	62	7	3.8%		12	2	3.6%										Other	10	5.5%		Other	2	3.6%
	75	3	1.6%		42	2	3.6%																
	81	2	1.1%		16	1	1.8%																
	85	2	1.1%		36	1	1.8%																

	54	1	0.5%		32	1	1.8%															
	64	1	0.5%		31	1	1.8%															
	65	1	0.5%		41	1	1.8%															
	71	1	0.5%		Total	55																
	74	1	0.5%																			
	84	1	0.5%																			
	Total	182																				
Crown-root fracture	51	8	53.3%		11	2	50.0%		Max	15	100.0%		Max	4	100.0%		I1	15	100.0%		I1	4	100.0%
	61	7	46.7%		21	2	50.0%		Mnd	0	0.0%		Mnd	0	0.0%							
	Total	15			Total	4																
Root fracture	51	7	46.7%		11	11	68.8%		Max	14	93.3%		Max	15	93.8%		I1	13	86.7%		I1	14	87.5%
	61	5	33.3%		21	2	12.5%		Mnd	1	6.7%		Mnd	1	6.3%		I2	1	6.7%		I2	2	12.5%
	53	1	6.7%		12	1	6.3%										C	1	6.7%		C	0	0.0%
	62	1	6.7%		22	1	6.3%										Other	0	0.0%		Other	0	0.0%
	71	1	6.7%		41	1	6.3%															
	Total	15			Total	16																
Enamel infraction	-	-	-		11	1	50.0%						Max	1	50.0%						I1	2	100.0%
					31	1	50.0%						Mnd	1	50.0%								
					Total	2																

I1, central incisor; I2, lateral incisor; C, canine; Max, maxilla; Mnd, mandible.

Appendix 2. Frequencies of TDIs according to week day.

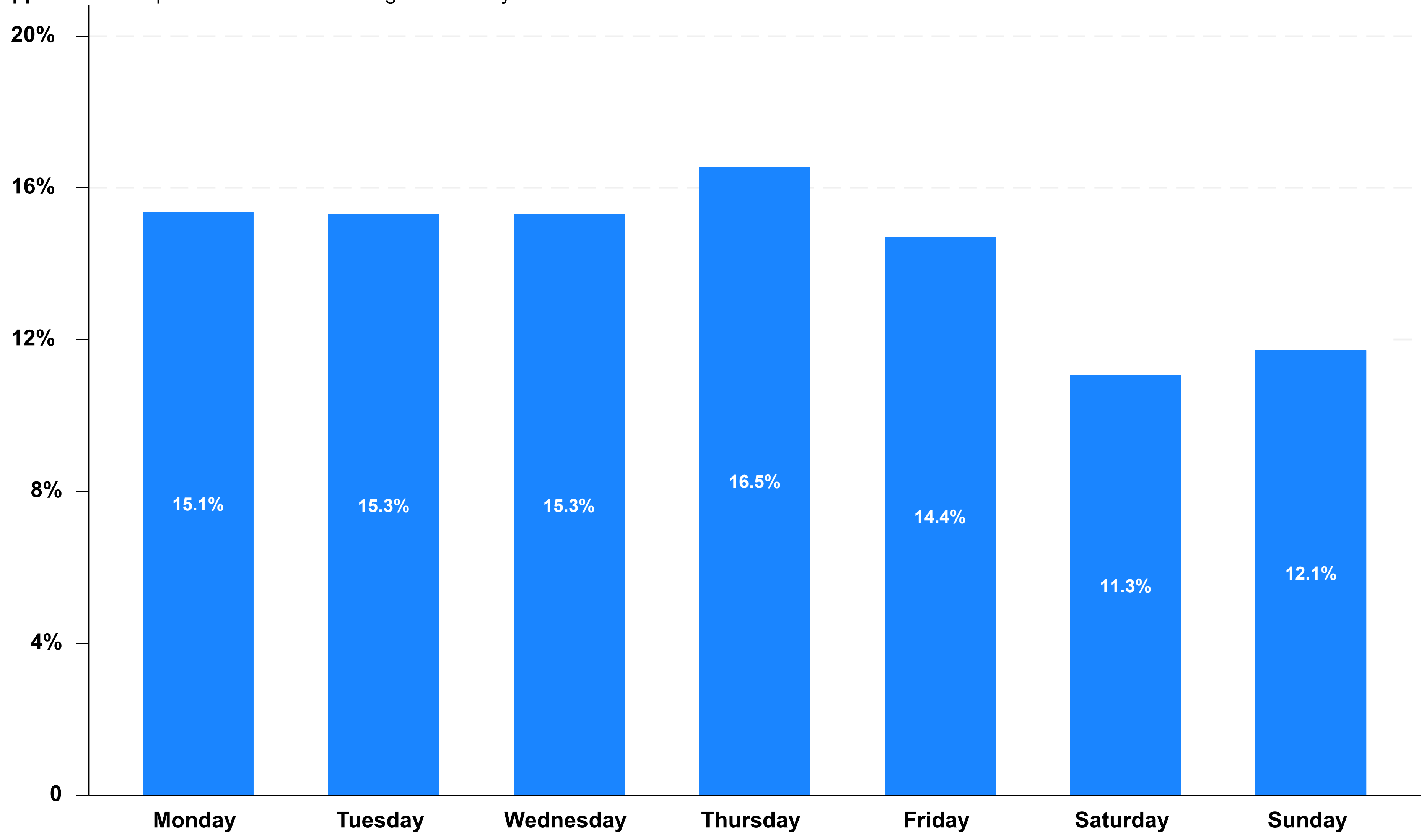

Appendix 3. Setting in which trauma occurred in both dentitions in %

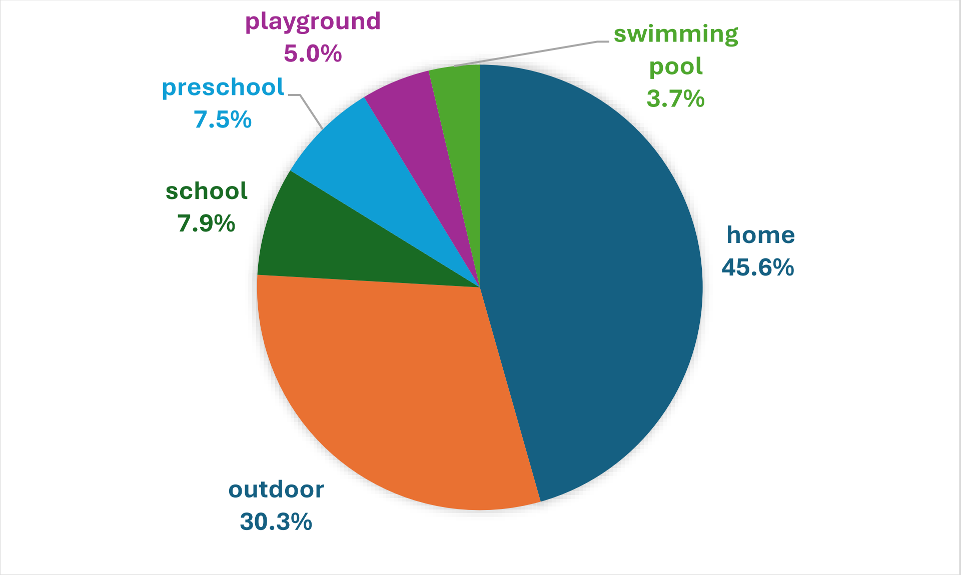

–

Appendix 4. Activities where trauma occurred in both dentitions in %

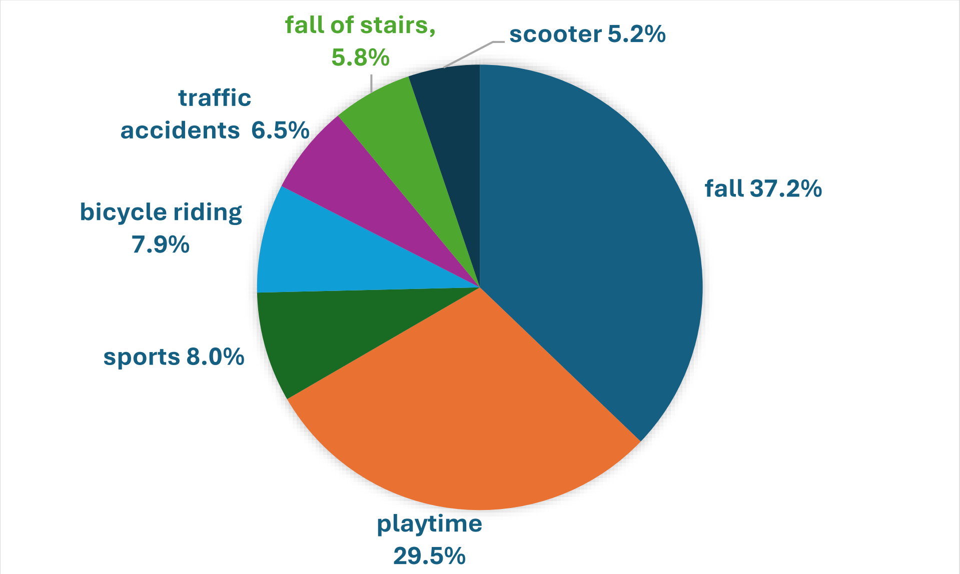

–
